# Influence of thermal maturity on carbazole distributions in coal source rocks during compaction pyrolysis experiments

**DOI:** 10.1038/s41598-024-57520-1

**Published:** 2024-03-21

**Authors:** Jian Bao, Yan Liu, Yunpeng Fan, Yaohui Xu, Kangle Ding, Zhigang Wen, Yang Li, Ye Gao, Cunyang Zhang, Lu Li

**Affiliations:** 1 Hubei Key Laboratory of Petroleum Geochemistry and Environment (Yangtze University), Wuhan, 430100 Hubei People’s Republic of China; 2 College of Resources and Environment, Yangtze University, Wuhan, 430100 Hubei People’s Republic of China; 3 College of Chemistry and Environmental Engineering, Yangtze University, Jingzhou, 434022 Hubei People’s Republic of China

**Keywords:** Carbazole compounds, Thermal maturity, Coal, Compaction pyrolysis experiment, Hydrocarbon migration, Solid Earth sciences, Geochemistry

## Abstract

Carbazole compounds are widely used in determining the direction of petroleum migration, but the effect of thermal maturity on carbazoles is still ambiguity. In this paper, using compaction pyrolysis simulation experiments, artificial mature samples with vitrinite reflectance (*R*_*o*_) range from 0.38 to 3.0% were acquired. And the content and composition change characteristics of carbazole compounds were analyzed in coal source rocks. The experimental results showed that thermal maturity controls the generation of a large amount of carbazole compounds in coal rocks. Compared with the low mature stage, the content of carbazole compounds was about 10–100 times higher in the mature stage. With the increasing maturity, in the coal sample, the content of carbazole compounds showed a trend of first increasing and then decreasing. In derivatives of carbazole, the corresponding maturity for the maximum generation of ethylcarbazole (EC), dimethylcarbazole (DMCA), methylcarbazole (MCA), carbazole (CA) and benzocarbazole (BCA) performed the increasing sequence. With the increasing maturity, the relative abundance of 2-MCA, 1,7-DMCA and benzo[a]carbazole increased with the increasing maturity, while 4-MCA, 1,4-DMCA and benzo[c]carbazole gradually decreased. Benzocarbazole ratio [a]/[a] +[ c] varies only in a narrow range 0.36–0.61 in the entire maturity range, suggesting limited maturity dependence. The experimental conclusion provides more theoretical basis for future geochemical analysis using carbazole compounds.

## Introduction

Organic nitrogen-containing compounds with heterocyclic aromatic structures are representative components of non-hydrocarbon in crude oil^1,2^. Heterocyclic aromatic nitrogen-containing compounds in petroleum can be divided into two categories: neutral pyrrole and basic pyridine compounds^3,4^. Carbazole and its derivatives, as important neutral pyrrole compounds, are effective molecular markers for tracking the migration distance and charging pathway of hydrocarbon, and their content and composition change regularly during oil and gas transportation processes. According to the migration fractionation mechanism of carbazole compounds, with the increase of migration distance, the total concentration of carbazole nitrogen-containing compounds gradually decreases, the relative content of shielded compounds increases, and the content of exposed compounds gradually decreases. Therefore, carbazole compounds are commonly used as indicators of petroleum migration, it has been widely used in petroleum geochemical research in the past 20 years^5–11^. However, the abundance and relative composition of nitrogen-containing compounds in crude oil and source rocks are not only affected by the migration fractionation effect, but also by various factors such as sedimentary facies and environment^12–15^, thermal maturity^16–25^ and biodegradation^26–31^. The understanding of the effect of thermal maturity on the content changes of carbazoles is still controversial. Some researchers believe that the effect is limited. For example, Li et al.^5^ believed in the early stage that primary and secondary oil migration had a great influence on the abundance and distribution of carbazole derivatives in reservoir oil, while organic matter source input, sedimentary environment and thermal maturity did not seem to be the main control factor. Hallmann et al.^32^ studied the distribution of carbazoles in raw oil in Gidgealpa oilfield, and believed that the distribution of benzocarbazole has no relationship with lithofacies, organic facies and maturity, but the composition of alkyl carbazole has obvious correlation with lithofacies and organic facies. There are also studies that the effect of thermal maturity is very significant^15,18–21,23–25^. For example, Li et al.^17^ investigated the carbazoles in the extracts of marine carbonate rocks with %*R*_*o*_ range of 0.45–1.30%, they found that both the total concentration of carbazoles and benzo[a]carbazole/(benzo[a]carbazole + benzo[c]carbazole) increase with the increasing of maturity within a certain maturity range. In shale samples from the Jurassic Posidonia in northern Germany, with the increasing maturity, the ratios of 1-methylcarbazole/(1-+ 2-methylcarbazole) and benzo[a]carbazole/(benzo[a]carbazole + benzo[c]carbazole) all increased^13^. Zhang et al.^21^ studied the distribution of carbazoles in lacustrine source rocks in the Qaidam Basin, suggested that the relative abundance of 1-MCA (methylcarbazole) showed a decreasing trend at immature stage and an increasing trend in the mature stage, while the relative abundance of the other three isomers (2-, 3- and 4-MCA) showed opposite changes, showing a “two-stage” change. Over the entire maturity range, the benzocarbazole ratio benzo[a]carbazole/(benzo[a]carbazole + benzo[c]carbazole) increased with maturity, but the ratio changed less, only between 0.52 and 0.61.

According to different research methods, the current research on the effect of thermal maturity on carbazole compounds is shown in Table 1. When analyzing natural samples of different depths and maturities to discuss the effect of thermal maturity on carbazole compounds^15,16,21,32^, it is difficult to reveal the effect of thermal maturity on carbazole compounds alone due to the combined effect of multiple factors such as sedimentary environment and biodegradation under geological conditions. In order to overcome the constraints of other influencing factors, thermal simulation experiments are usually used to exclude the influence of other factors. For example, the pyrolysis simulation experiment of the monomer compound carbazole revealed the changing patterns of carbazole formation and pyrolysis^33,34^. Cleeg et al.^18^ and Chen et al.^22^ conducted hydrous pyrolysis experiments in a closed system to analyze the effect of pyrolysis temperature on carbazole compounds in limestone and coal. They found that within a certain temperature range, the content of carbazole compounds increased with the increase of pyrolysis temperature. However, due to the relatively low temperature at which researchers conducted hydrous pyrolysis experiments, a complete maturity sequence could not be obtained, and the influence of thermal maturity on carbazole compounds could not be fully revealed.

**Table 1:** Experimental comparison of the effect of thermal maturity on carbazole compounds.

Research method	Thermal simulation experiment	System type	Fluid	Overlying static rock pressure	Main features	References
Natural sample research	No	/	No	No	The experimental data is closer to the results under real geological conditions, but the geological conditions are complex, and other factors such as sedimentary environment, biodegradation, and the source of organic matter cannot be ruled out	Li et al.^12^, Zhang et al.^15^, Harrison et al.^16^, Hallmann et al.^32^
Hydrous pyrolysis experiments	Yes	Closed system	Liquid water	No	This experiment can exclude factors other than thermal maturity, but the hydrous pyrolysis experiments conducted by the researchers reached a relatively low temperature and could not obtain a more complete maturity sequence	Cleeg et al.^18^, Chen et al.^22^
Compaction pyrolysis experiment	Yes	Semi open system	Liquid water	0.1-270Mpa	This system instrument not only considers the influence of temperature on hydrocarbon generation, but also takes into account other factors such as pressure, fluid medium, and pore space. The experimental conditions are closer to geological conditions, and the simulated temperature of the experiment can reach a higher level	This paper

Based on the above reasons, on the basis of previous pyrolysis simulation experiments^18,22,33,34^, this article adopts a semi open system pyrolysis experimental device that is closer to geological conditions. Selecting low maturity coal rocks for experiments, a relatively complete maturity sequence from 0.38%*R*_*o*_ to 3.02%*R*_*o*_ was obtained. This article aims to explore the formation and transformation laws of carbazole in the thermal evolution process by analyzing the influence of thermal maturity on the distribution of carbazole in coal, and provide more theoretical basis for the application of carbazole compounds in oil and gas migration.

## Samples and experimental

### Samples

The Longkou lignite samples were used for compaction pyrolysis experiment in this study, and the basic geochemical information of the experimental samples is shown in Table 2.

**Table 2:** Basic geochemical information of sample.

Sample number	Sample type	Sample formation	TOC (%)	S_1_ (mg/g)	S_2_ (mg/g)	T_max_ (°C)	HI (mg/gTOC)	*R*_*o*_ (%)
HXHM	Lignite	E_1_h	78.5	3.5	274.8	431	350	0.38

### Experimental

#### Compaction pyrolysis experiment

**Figure 1:**
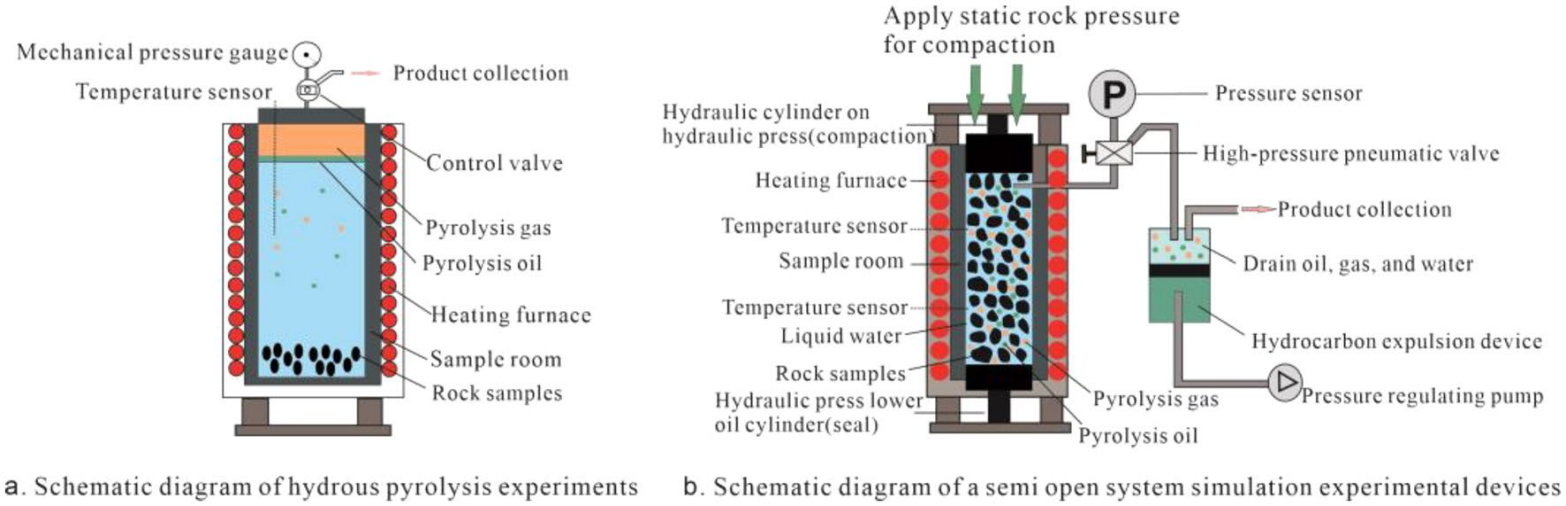
Schematic diagram of simulation experimental devices for closed and semi open systems (Bottom image from He et al.^37^).

The thermal simulation experiment for this study was completed at the Key Laboratory of Petroleum Geochemistry and Environment, Yangtze University. The experimental equipment adopts the DK-III formation pore thermal pressure hydrocarbon generation and expulsion simulation experimental instrument developed by Wuxi Institute of Petroleum Geology, Sinopec^35–37^. The schematic diagram of the device structure is shown in Fig. 1b. Compared to the hydrous pyrolysis experiments used by Cleeg et al.^18^ and Chen et al.^22^ (Fig. 1a), this system instrument not only considers the influence of temperature on hydrocarbon generation, but also takes into account other factors such as pressure, fluid medium, and pore space. At the same time, it can also link and control the generation, discharge, and retention processes of oil and gas, thereby achieving integrated simulation of oil and gas generation, discharge, and retention, to better approximate the process of hydrocarbon generation and expulsion under actual geological conditions^35^.

The experimental steps are as follows: Firstly, divided the coal sample into 11 equal parts, each with a mass of approximately 15 g, and pressed them into a special sample compartment at a pressure of not less than 5 Mpa. Loaded the entire sample compartment into the reaction vessel. The temperature settings for the pyrolysis experiment are 320 °C, 340 °C, 360 °C, 400 °C, 414 °C, 440 °C, 460 °C, 480 °C, 500 °C, 550 °C and 600 °C. The heating procedure is as follows: first raised from room temperature to 150 °C for 30 min, then raised to the corresponding temperature point at a heating rate of 20 °C/h, and then stopped heating. During the experiment, a semi-open system was used to control the hydrocarbon discharge pressure to 30 Mpa. When the pressure was exceeded, it was automatically discharged into the product collector. After the heating was stopped, the temperature of the kettle body dropped to 120 °C. Opened the hydrocarbon discharge valve and completely discharge the high-pressure fluid in the kettle until the pressure in the kettle dropped to atmospheric pressure. Opened the kettle body and remove the experimental residue. Divided the residual coal sample into three parts, one for Soxhlet extraction, one for vitrinite reflectance measurement, and one for other basic tests. For more detailed experimental steps and equipment schematic diagrams, please refer to the literature^36,38^.

#### Vitrinite reflectance

Vitrinite reflectance (*R*_*o*_) were performed on a Zeiss Axio Scope A1/J&M Msp200 digital maceral analysis system. The coal source rock was cut into blocks with a length of about 2 cm and a width of about 2 cm, and the samples were consolidated with epoxy resin to make a piece of light sheet, which was polished on a Buehler automatic grinding and polishing machine to obtain a smooth surface for microscopic examination. Each temperature point corresponding to the number of sample measuring points is not less than 30, with the normal distribution of discrete results of the mathematical average value of the temperature point represents the *R*_*o*_ value, used to calibrate the thermal maturity stage of each temperature point.

#### Soxhlet extraction and separation

Crushed approximately 10 g of pyrolysis residual coal sample into powder with less than 120 mesh size. Loaded into a Soxhlet extractor, extracted with CH_2_CL_2_ for 72 h, and obtained chloroform asphalt “A”. The N-containing fractions were isolated using the method reported by Li et al.^5,39^.

#### GC–MS

In order to quantify the concentration of carbazole, 9-phenylcarbazole was added as an internal standard before GC–MS analysis, and the concentration of carbazole compounds was quantified by internal standard method, and its content was calculated. Gas chromatography-mass spectrometry (GC–MS) analysis of the pyrrolic nitrogen fractions was carried out using an HP 7890 gas chromatograph equipped with an HP 5975 mass selective detector. A fused silica capillary column (30 m × 0.25 mm) coated with HP-5MS (film thickness 0.25 um) was used with He as carrier gas at a constant flow of 1.0 ml min^−1^. The gas chromatograph oven temperature was programmed from 50 to 100 °C at 20 °C min^−1^, then to 315 °C at 4 °C min^−1^. The mass spectrometer was operated in multiple ion detection (MID) mode.

Qualitative analysis of carbazole (CA), methylcarbazole (MCA), dimethylcarbazole (DMCA), ethylcarbazole (EC), and benzocarbazole (BCA) were based on literature^12^. The absolute content of carbazole compounds and their derivatives were calculated by the following formula: Compound content (μg/g) = (area of compound peak/area of internal standard peak) × weight of standard sample (μg)/weight of sample (g).

## Results and discussion

### Basic experimental data

**Figure 2:**
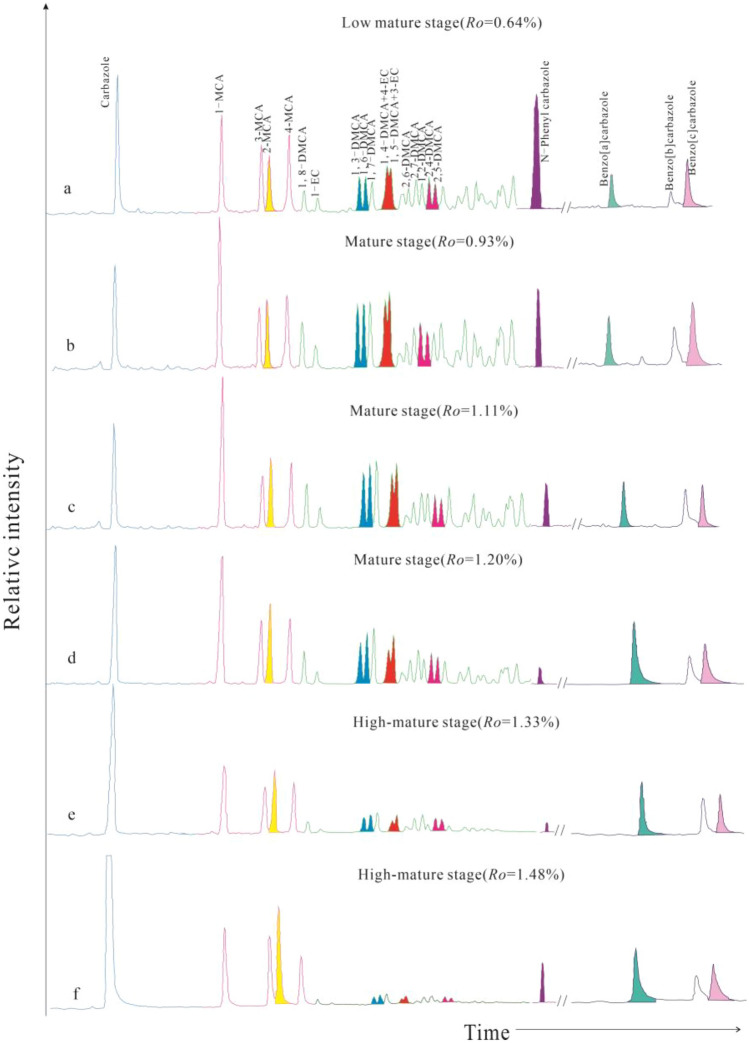
Single ion monitoring (m/z 167,181,195,217) chromatograms showing the distributions of carbazole, C_1_-carbazoles, C_2_-carbazoles and benzocarbazoles at different maturity levels.

The content of carbazole compounds in coal source rocks at the different pyrolysis temperature was shown in Table 3. Carbazole, methylcarbazole, dimethylcarbazole, ethylcarbazole and benzocarbazole are the main carbazole compounds. Figure 2 showed the distribution characteristics of carbazoles at different maturity levels.

**Table 3:** The content of carbazole and its derivatives in coal at the different pyrolysis temperature (μg/g).

	Temperature (℃)	320	340	360	400	414	440	460	480	500	550	600
	*R*_*o*_ (%)	0.60	0.64	0.68	0.93	1.11	1.20	1.33	1.48	1.58	2.26	3.03
Ion	Name											
167	CA	19.40	27.27	22.38	28.25	64.05	420.08	1,700.96	6,686.10	4,307.95	25.95	3.67
181	1-MCA	13.38	17.55	15.08	38.95	94.46	421.05	691.45	744.85	610.25	2.75	0.99
181	3-MCA	8.98	12.24	10.13	15.22	32.97	177.36	488.19	641.76	495.01	1.93	0.66
181	2-MCA	8.55	11.62	9.72	16.98	40.81	222.26	615.65	986.31	710.94	3.77	1.35
181	4-MCA	12.14	16.39	14.41	20.18	40.24	185.70	449.23	505.24	397.64	1.75	1.69
195	1,8-DMCA	2.60	3.70	3.24	9.82	22.68	68.89	76.94	45.05	36.80	0.43	/
195	1-EC	1.50	1.94	1.84	4.71	8.62	19.53	17.32	12.53	9.43	0.14	/
195	1,3-DMCA	3.89	5.03	4.12	12.31	27.50	94.75	105.77	63.41	55.31	0.60	/
195	1,6-DMCA	4.59	5.76	4.69	14.03	32.26	113.31	132.18	80.26	70.13	0.75	/
195	1,7-DMCA	3.82	5.27	4.24	14.58	35.39	123.22	153.80	108.78	86.62	1.08	/
195	1,4-DMCA + 4-EC	7.85	9.40	7.93	14.66	30.56	74.12	78.02	41.55	32.91	0.28	/
195	1,5-DMCA + 3-EC	5.45	7.74	6.83	14.91	31.28	102.49	128.07	76.52	59.86	0.71	/
195	2,6-DMCA	3.08	4.00	3.46	6.47	13.63	60.58	109.78	69.78	57.66	0.61	/
195	2,7-DMCA	4.81	6.10	5.88	8.88	19.03	81.72	138.62	73.67	65.58	0.62	/
195	1,2-DMCA	3.32	4.99	3.80	9.86	17.08	47.62	49.74	33.73	22.22	0.24	/
195	2,4-DMCA	3.76	4.89	4.11	7.90	16.75	59.45	101.53	57.89	48.10	0.56	/
195	2,5-DMCA	3.63	4.46	3.92	7.69	15.73	59.45	98.33	58.49	47.20	0.55	/
195	2,3-DMCA	2.18	2.82	2.46	4.09	6.94	22.41	31.72	17.74	15.33	0.19	/
195	3,4-DMCA	2.55	3.14	3.00	3.86	6.01	16.33	18.96	9.40	7.32	0.15	/
217	B[a]CA	1.30	1.93	1.57	2.56	3.12	14.93	57.68	158.82	109.16	2.49	/
217	B[b]CA	0.67	1.17	0.98	1.91	2.87	13.01	45.96	75.37	57.32	0.95	/
217	B[c]CA	2.34	2.96	2.58	3.01	3.34	13.21	49.08	121.29	79.21	1.58	/
	CA	19.40	27.27	23.38	28.25	64.05	420.08	1700.96	6686.10	4307.95	25.95	3.67
	MCA	43.05	57.80	49.34	91.33	208.48	1066.37	2244.52	2878.16	2213.84	10.60	4.69
	DMCA	51.52	67.32	57.68	129.06	274.85	924.33	1233.47	736.25	605.06	6.77	/
	BCA	4.31	6.06	5.13	7.48	9.32	41.55	152.72	355.48	245.69	5.02	/
	EC	1.50	1.94	1.84	4.71	8.62	19.53	17.32	12.53	9.43	0.48	/
	AC + BC	119.7	160.3	136.3	260.83	565.32	2,411.87	5,338.99	10,668.5	7,381.97	48.42	8.36
	1-MCA/MCA	0.31	0.30	0.31	0.43	0.45	0.42	0.34	0.26	0.28	0.27	/
	2-MCA/MCA	0.20	0.20	0.20	0.19	0.20	0.22	0.27	0.34	0.32	0.37	/
	3-MCA/MCA	0.21	0.21	0.21	0.17	0.16	0.18	0.22	0.22	0.22	0.19	/
	4-MCA/MCA	0.28	0.28	0.29	0.22	0.19	0.18	0.20	0.18	0.18	0.17	/
	B[a]CA/BCA	0.30	0.32	0.31	0.34	0.33	0.36	0.38	0.45	0.44	0.50	/
	B[b]CA/BCA	0.15	0.19	0.19	0.26	0.31	0.32	0.30	0.21	0.23	0.19	/
	B[c]CA/BCA	0.54	0.49	0.50	0.40	0.36	0.32	0.32	0.34	0.32	0.31	/

*CA* carbazole, *MCA* methylcarbazole, *DMCA* dimethylcarbazole, *EC* ethylcarbazole, *BCA* Benzocarbazole, *B[a]CA* benzo[a]carbazole, *B[b]CA* benzo[b]carbazole, *B[c]CA* benzo[c]carbazole, *AC* + *BC* alkylcarbazole + benzocarbazole.

#### Carbazole compounds

There is a close relationship between the content of carbazoles and the maturity in coal source rocks. With the increasing maturity, the content of carbazoles showed a trend of first increasing and then decreasing. In the low mature stage of *R*_*o*_ < 0.7%, the total amount of carbazole compounds increased slowly with the increasing maturity, and the total content was less than 160 μg/g (Table 3). From 0.7%*R*_*o*_, the total content of carbazole compounds rapidly increased during the mature stage (0.7% < *R*_*o*_ < 1.3%) and high mature stage (1.3% < *R*_*o*_ < 2.0%), and reached its maximum (10,668.5 μg/g) at 1.48%*R*_*o*_. With the maturity continued to increase, the total content of carbazole compounds began to decrease rapidly after reaching the maximum. In the over-mature stage (*R*_*o*_ > 2.0%), the total content of carbazole compounds was only 48.42 μg/g at 2.26% *R*_*o*_. In the stage of *R*_*o*_ > 3.0%, carbazole compounds were hardly detected in the samples.

**Figure 3:**
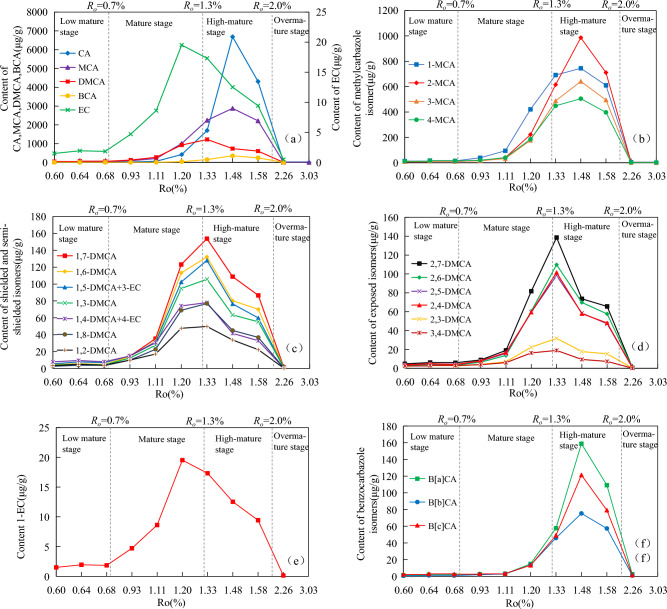
Content of carbazoles and their isomers with the increasing maturity. (**a**) carbazole compounds. (**b**) methylcarbazole isomers. (**c**,**d**) dimethylcarbazole isomers. (**e**) ethylcarbazole. (**f**) benzocarbazole isomers.

The content of various derivatives of carbazole compounds also exhibited similar changes, including carbazole (Fig. 3a), methylcarbazole isomers (Fig. 3b), dimethylcarbazole isomers (Fig. 3c,d), ethylcarbazole (Fig. 3e), and benzocarbazole isomers (Fig. 3f). In the low mature stage, the content of carbazole, methylcarbazoles, dimethylcarbazoles, ethylcarbazole and benzocarbazoles were all lower than 70 μg/g (Fig. 3a). Among all the carbazole compounds, dimethylcarbazoles and ethylcarbazole showed the highest (maximum 67.32 μg/g) and lowest (lower than 2 μg/g) content, respectively. Ethylcarbazole, dimethylcarbazoles and methylcarbazoles were rapidly generated in the mature stage, with maximum values of 19.53 μg/g (1.2%*R*_*o*_), 1233.47 μg/g (1.33%*R*_*o*_) and 2878.16 μg/g (1.48%*R*_*o*_), respectively. Carbazole and benzocarbazoles were rapidly generated in the high mature stage, and both reached the maximum values at 1.48%*R*_*o*_, and the corresponding maximum of their content were 6686.10 μg/g and 355.48 μg/g, respectively. Cleeg et al.^18^ discovered that the concentrations of carbazole, methylcarbazoles, C_2_-carbazoles, benzo[a]carbazole and benzo[c]carbazole increase signicantly and eventually reach a maximum at 360 °C (1.4% *R*_*o*_) during the hydration and pyrolysis of organic rich source rocks in the Ghareb Formation (Upper Cretaceous, Jordan). With the increasing maturity, the content of carbazole compounds rapidly decreased after reaching their maximum. At 2.26%*R*_*o*_, except for carbazole, with content of 25.95 μg/g, the content of other carbazole compounds were below 10 μg/g. The content of ethylcarbazole and dimethylcarbazoles began to decrease after reaching the maximum earlier. Thermal stress may cause the alkyl side chain to crack, and even converted into methylcarbazoles or the product of intramolecular rearrangement. From the perspective of chemical stability, the more and longer the branches connected by carbon sites, the more likely to break. The carbazole content increased sharply at 1.33%*R*_*o*_, indicating that the carbazole may be partially derived from the product formed by the demethylation of ethylcarbazole and dimethylcarbazoles. When the content of carbazole compounds reached its maximum, the content showed a trend of carbazole > methylcarbazoles > dimethylcarbazoles > benzocarbazoles > ethylcarbazole, alkylcarbazoles > alkylbenzocarbazoles. However, both dimethylcarbazoles and methylcarbazoles were higher than carbazole in the early mature stage (*R*_*o*_ < 1.20%), which may be a result of the initial exfoliation of hydrocarbon in the samples rather than related to thermally-induced selective alkylation and dealkylation.

The change law of the content of carbazole compounds depends on the rate of formation and cleavage of carbazole compounds with the increasing maturity. When the formation rate is higher than the cleavage rate, the content increases, otherwise it decreases. The main formation stage of carbazoles is later than that of hydrocarbon generation, but its formation curve is close to that of hydrocarbon generation. The content of carbazole compounds is relatively low in the low mature stage, and rapidly increases in the mature and high mature stages. However, carbazole compounds are almost undetectable in coal source rocks after the over mature stage. It can be seen that carbazole compounds may not be completely inherited from organic sediments, and their content increases significantly with the appearance of the main hydrocarbon generation period. According to the theory of kerogen oil generation, under certain thermal maturation conditions, cracking can occur and oil generation can begin. Therefore, carbazole nitrogen-containing compounds may also be based on a certain degree of maturity, and they may also arise from cracking. This article argues that maturation plays a key role in controlling carbazole distributions in coal source rocks.

#### C_1_-carbazoles

**Figure 4:**
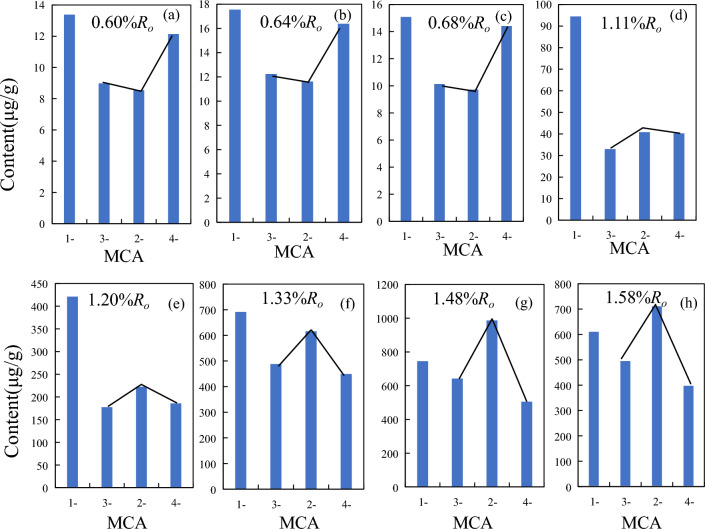
Content distribution characteristics of methylcarbazole isomers with the increasing maturity.

In addition to carbazole, methylcarbazoles is also the main component of carbazole compounds. Methylcarbazoles can be divided into four isomers according to the different conditions of carbon site substitution by methyl, namely 1-MCA, 2-MCA, 3-MCA and 4-MCA. The GC/MS data of carbazole compounds showed that the content of methylcarbazole isomers also changed regularly with the increasing maturity (Fig. 3b). In the low mature stage, the content of the four methylcarbazole isomers were all below 20 μg/g, with 1-methylcarbazole as the highest(17.55 μg/g), followed by 4-MCA, 3-MCA and 2-MCA. The distribution characteristics of 2-, 3-, and 4-MCA isomers showed an asymmetric "∨" type in the low mature stage (Fig. 4). In the mature stage, the content of methylcarbazoles increased rapidly, with 1-MCA (38–680 μg/g) and 2-MCA (16–600 μg/g) increasing rapidly. The content of 1-MCA had an advantage at the stage of *R*_*o*_ < 1.3%, while the content of 4-MCA gradually decreased and became the lowest. After entering the high mature stage, 2-MCA generated at a faster rate, and the content gradually exceeded 1-MCA. At this time, the distribution characteristics of the three isomers of 2-, 3-, and 4-MCA were asymmetric “∧” type (Fig. 4). The content of the four methylcarbazole isomers all reached the maximum at 1.48%*R*_*o*_. Among the methylcarbazole isomers, the content of 2-MCA was the highest (maximum 986.31 μg/g), followed by the content of 1-MCA (744.85 μg/g), and the content of 4-MCA was lowest (505.24 μg/g). With the maturity continued to increase, the content of the four isomers decreased rapidly at the stage of *R*_*o*_ > 1.48%, and the content of the four isomers were all lower than 4 μg/g at 2.26%*R*_*o*_. The methylcarbazole isomers were almost undetectable at 3.0%*R*_*o*_. Zhang et al.^21^ studied the Tertiary source rocks in the salt lacustrine facies environment of the Qaidam Basin, suggesting that 1-MCA in methylcarbazoles is the isomer with the highest content among the four isomers regardless of maturity. Li et al.^5^ believed that 1-MCA is the isomer with the lowest content and 4-MCA is the isomer with the highest content in the shale from Liaohe Basin. Source rock type and depositional environment have a great influence on the content and composition distribution characteristics of methylcarbazoles.

**Figure 5:**
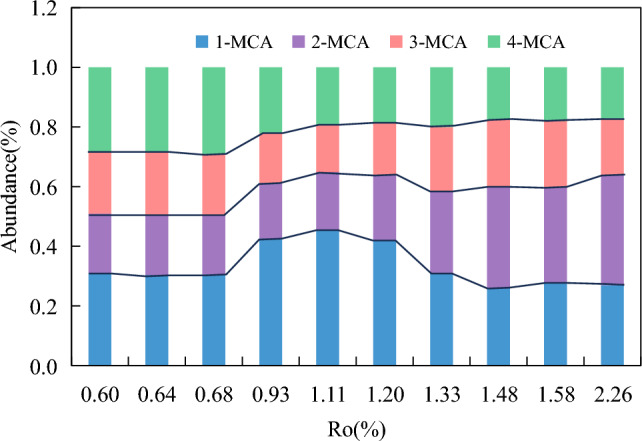
Changes of relative abundance of methylcarbazole isomers with the increasing maturity.

With the increasing maturity, the relative abundance of 2-MCA gradually increased from 19 to 37%, with a large change range. The relative abundance of 4-MCA gradually decreased from 28 to 17% (Fig. 5). The relative abundance of 1-MCA gradually increased from 31 to 45%, then gradually decreased to 27%, and the maximum relative abundance appeared at 1.11%*R*_*o*_. The relative abundance of 3-MCA varied only between 16 and 22%, and the variation range was relatively small (Table 3).

#### C_2_-carbazoles

Dimethylcarbazoles can be divided into shielded isomers (the H atoms at the 1-carbon position and the 8-carbon position are substituted by alkyl groups, referred to as G1 type), semi-shielded isomers (only the H atoms at the 1-carbon position are substituted by alkyl groups, referred to as G2 type), and exposed isomers (the H atoms at the 1-carbon position and the 8-carbon position are not substituted by alkyl groups, referred to as G3 type) based on the position of carbon substituted by alkyl groups.

**Figure 6:**
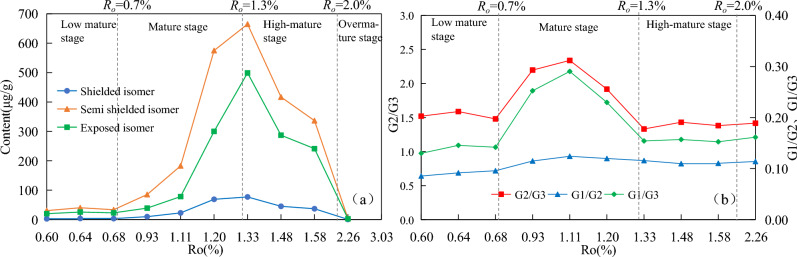
Changes in total content and ratios of dimethylcarbazole isomers with the increasing maturity. (**a**) total content of dimethylcarbazole isomers. (**b**) ratio of dimethylcarbazole isomers.

With the increasing maturity, the total content of shielded isomers, semi-shielded isomers and exposed isomers all first increasing and then decreasing (Fig. 6a). In the low mature stage, the total content of the three types of isomers were all lower than 40 μg/g. After entering the mature stage, the content of the three isomers increased rapidly, and the content all reached the maximum at 1.33%*R*_*o*_ (maximum 76.94 μg/g, 664.90 μg/g and 498.95 μg/g, respectively). With the maturity continued to increase, the content of the three isomers began to decrease rapidly, and the content of the three isomers were all below 4 μg/g at 2.26%*R*_*o*_. The content of semi shielded isomers and shielded isomers were the highest and lowest within the entire maturity range. The exposed isomers show higher abundance compared to the shielded isomers, which is consistent with the thermodynamic equilibrium principle. From a chemical perspective, the chemical stability of the exposed isomer is higher than that of the shielded isomers.

In the dimethylcarbazoles, the ratio of shielded isomers to semi-shielded isomers content remained essentially unchanged, varying only between 0.09 and 0.12 over the entire maturity range (Fig. 6b). G1/G3 and G2/G3 showed a large change in the maturity stage, which first increasing and then decreasing, and reached maximum at 1.11%*R*_*o*_.

The content of each monomer isomer of dimethylcarbazoles was lower than 10 μg/g in the low mature stage (Table 3), and all increased rapidly in the mature stage. Except that the content of 1-EC reached the maximum at 1.20%*R*_*o*_, the rest of the dimethylcarbazole isomers are either exposed isomers (2,6-DMCA and 2,4-DMCA), shielded isomers (1,8-DMCA), or semi-shielded isomers (1,7-DMCA and 1,3-DMCA), with maximum at 1.33%*R*_*o*_. At this stage, the content of 1,7-DMCA is the highest (153.80 μg/g) and the content of 3,4-DMCA is the lowest (18.96 μg/g) (Table 3). With the maturity continued to increase, the content of dimethylcarbazole isomers decreased rapidly. The content of each dimethyl isomer is below 1 μg/g, and dimethylcarbazole isomers were almost undetectable in the samples at 2.26%*R*_*o*_ (Fig. 3c–e).

Over the entire maturity range, the content of 1,7-DMCA, 1,6-DMCA, and 2,6-DMCA were higher than those of 1,2-DMCA, 1,3-DMCA, and 2,3-DMCA replaced by symmetric carbon sites, while the content of 2,4-DMCA and 2,5-DMCA were basically the same (Table 3). 1,7-DMCA and 2,7-DMCA existed with the highest abundance in semi-shielded and exposed isomers, respectively, followed by 1,6-DMCA and 2,6-DMCA. However, those isomers with adjacent substituents such as 1,2-DMCA, 2,3-DMCA, and 3,4-DMCA were always detected with less abundance. The general distribution of C_2_-carbazole is similar to the reported studies^40,42^.

**Figure 7:**
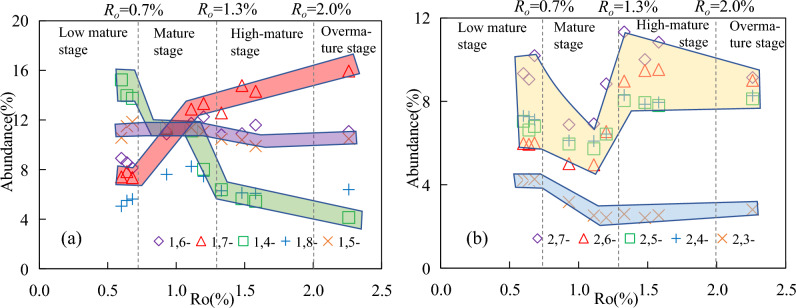
Changes in relative abundance of dimethylcarbazole isomers with the increasing maturity. (**a**) shielded and semi-shielded isomers. (**b**) exposed isomers.

With the increasing maturity, the relative abundance changes of the semi-shielded isomers and the exposed isomers were completely different. The relative abundance of 1,7-DMCA gradually increased from 7 to 16%. The relative abundance of 1,4-DMCA gradually decreased from 15 to 4%. The relative abundance of 1,5-DMCA is between 9 and 11% over the entire maturity range, and remains basically unchanged. The relative abundance of 1,8-DMCA showed a significant increasing and then decreasing trend during the mature stage, with maximum (8.25%) at 1.11%*R*_*o.*_ However, the relative abundance remains basically unchanged after 1.5%*R*_*o*_ (Fig. 7a). The relative abundance of 2,4-DMCA, 2,5-DMCA, 2,6-DMCA, and 2,7-DMCA showed a significant decrease and then increase during the mature stage, and the minimum also appeared at 1.11%*R*_*o*_. Their relative abundance remained basically unchanged after 1.5%*R*_*o*_. The relative abundance of 2,3-DMCA gradually decreased from 4.23 to 2.53% (Fig. 7b).

**Figure 8:**
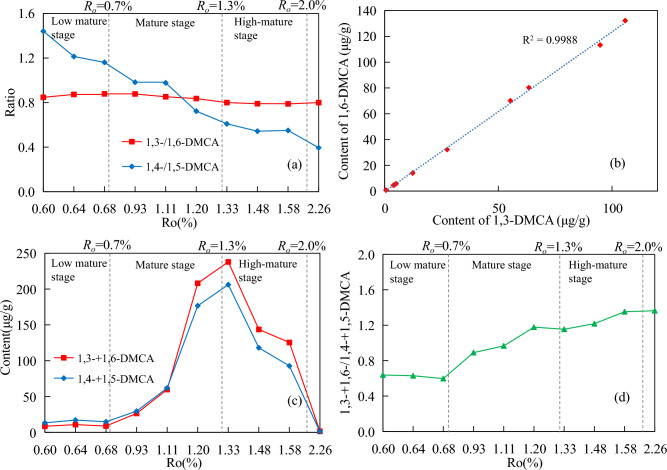
Relationship diagram between the content and ratios of semi-shielded isomers and maturity. (**a**) 1,3-/1,6-DMCA and 1,4-/1,5-DMCA. (**b**) 1,3-DMCA and 1,6-DMCA. (**c**) 1,3-+1,6-DMCA and 1,4-+1,5-DMCA. (**d**) 1,3-+1,6-/1,4-+1,5-DMCA.

Over the entire maturity range, 1,3-/1,6-DMCA remains basically unchanged, ranging between 0.79 and 0.88 (mean value of 0.83, the standard deviation of 0.03) (Fig. 8a). Zhang et al.^43^ found that the neutral nitrogen-containing compounds in crude oil from the Pearl River Mouth Basin also had a similar situation.

The positive correlation between the content of 1,3-DMCA and 1,6-DMCA is shown in Fig. 8b, with R^2^ of 0.9988, and showing no change with the increasing maturity. This shows two points: ①1,3-DMCA and 1,6-DMCA may come from a common precursor; ②1,3-DMCA and 1,6-DMCA form in the same mechanism and at the same rate. Considering the structural characteristics of 1,3-DMCA and 1,6-DMCA, the kerogen matrix with the structural characteristics of 1-methylcarbazole is obviously the most likely to be the common precursor of both^42^. That is, a formation mode of 1,3-DMCA and 1,6-DMCA is formed by the methylation of 1-methylcarbazole at the 3,6 carbon sites, respectively. Theoretically, 1,4-/1,5-DMCA should exhibit the same characteristics as 1,3-/1,6-DMCA. However, over the entire maturity range, 1,4-/1,5-DMCA gradually decreased from 1.44 to 0.39, which was greatly affected by maturity. This may be due to the co-elution of 3-EC and 4-EC at the 5-carbon and 4-carbon positions. As shown in Fig. 2, the content of 1,4-DMCA is higher than that of 1,5-DMCA, and the content of 1,4-+1,5-DMCA is higher than that of 1,3-+1,6-DMCA in the early mature stage. With the increasing maturity, the content of 1,5-DMCA has a stronger advantage over 1,4-DMCA, while the content of 1,3-+1,6-DMCA has a stronger advantage over 1,4-+1,5-DMCA (Fig. 8c). The ratio of 1,3-+1,6-DMCA/1,4-+1,5-DMCA gradually increases from 0.64 to 1.36. (Fig. 8d).

**Figure 9:**
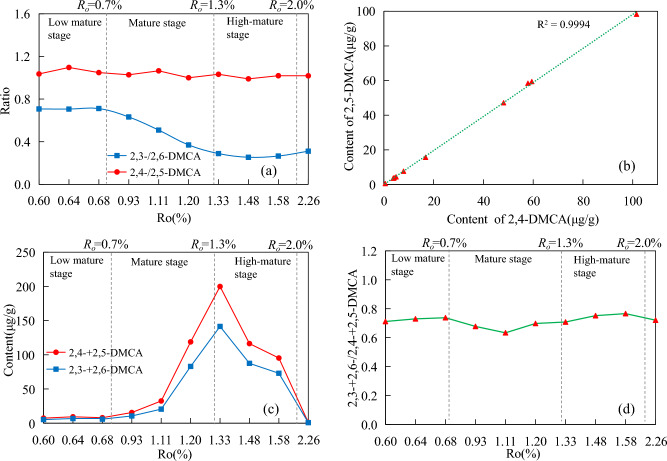
Relationship diagram between content and ratios of exposed isomers and maturity. (**a**) 2,3-/2,6-DMCA and 2,4-/2,5-DMCA. (**b**) 2,4-DMCA and 2,5-DMCA. (**c**) 2,3-+2,6-DMCA and 2,4-+2,5-DMCA (**d**) 2,3-+2,6-/2,4-+2,5-DMCA.

The exposed isomers exhibited a different variation pattern from the semi-shielded isomers. The relative abundance between 2,4-DMCA and 2,5-DMCA remained almost unchanged with the increasing maturity, with the 2,4-/2,5-DMCA ratio ranging between 0.99 and 1.10, with a mean of 1.03 (≈ 1) (Fig. 9a). The ratio did not change due to changes in the content and maturity of the two isomers.

**Figure 10:**
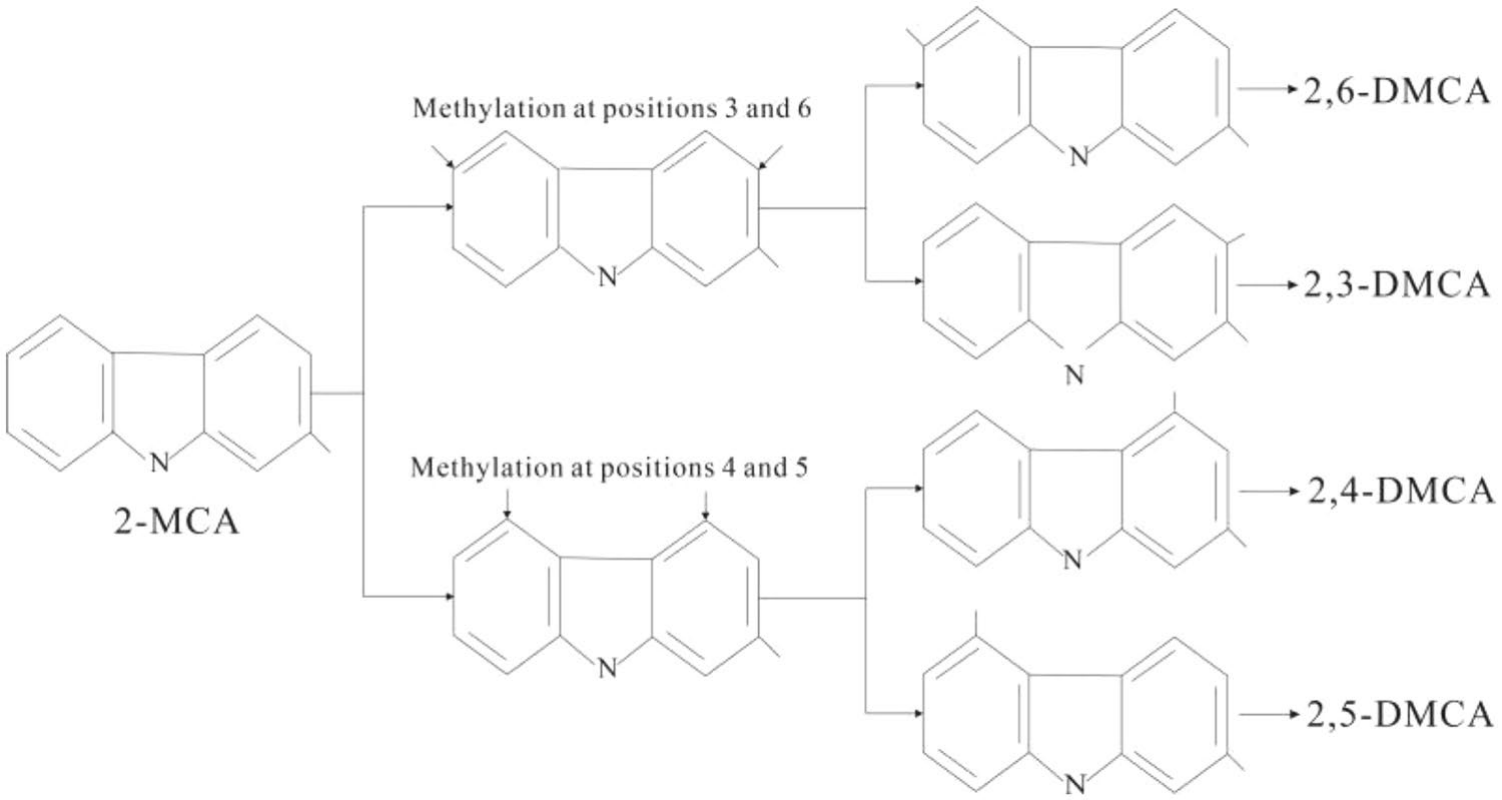
A formation mode of 2, X-dimethylcarbazole (modified after Zhang et al.^41^).

Figure 9b showed the correlation between the content of 2,4-DMCA and 2,5-DMCA (R^2^ = 0.9994), and 2,4-/2,5-DMCA also did not change with the increasing maturity. Similarly, 2,4-DMCA and 2,5-DMCA were formed at the same rate, based on the same formation mechanism and a common precursor. Kerogen matrices with 2-methylcarbazole are most likely to be the precursors of the two, namely, a formation mode of 2,4-DMCA and 2,5-DMCA is formed by methylation of 2-MCA at the 4-carbon and 5-carbon sites, respectively (Fig. 10). 2,4-/2,5-DMCA is almost 1 throughout the entire maturity range, indicating the consistency of 4-carbon and 5-carbon methylation in the further methylation process of 2-MCA, and the consistency of 4-carbon and 5-carbon chemical activity^42^.The relative abundance of ortho-substituted 2,3-DMCA was always relatively low over the entire maturity range, so the 2,3-/2,6-DMCA ratio decreased with the increasing maturity, indicated that the content of 2,6-DMCA was more dominant than 2,3-DMCA (Fig. 9c). 2,3-+2,6-/2,4-+2,5-DMCA only varied in a small range of 0.63–0.75, which indicated that 2,4-+2,5-DMCA was more dominant than 2,3-+2,6-DMCA over the entire maturity range (Fig. 9d). The inconsistency of their contents may indicate that 2-MCA has a competitive reaction mechanism at the 3- and 6-carbon, 4-carbon and 5-carbon sites during further methylation.

#### Benzocarbazoles

Most studies have shown that linear benzo[a]carbazole and subspherical benzo[c]carbazole typically exist in high abundance in benzocarbazole isomers, while benzo[b]carbazole is usually detected in low abundance. Except for coal samples containing high abundance of benzo[b]carbazole, the distribution of benzocarbazole isomers in most samples is similar^16,44^.

**Figure 11:**
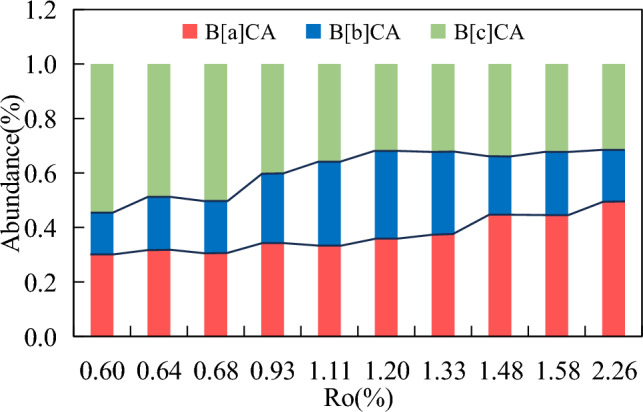
Changes of relative abundance of benzocarbazole isomers with the increasing maturity.

Coincidentally, with the increasing maturity, the content of the three benzocarbazole isomers showed a pattern of first increasing and then decreasing (Fig. 3f). In the low mature stage, the content of the three benzocarbazole isomers was all lower than 3 μg/g, the content of benzo[c]carbazole was slightly higher than that of benzo[a]carbazole, and the content of benzo [b] carbazole was the lowest (1.17 μg/g). With the increasing maturity, benzo[a]carbazole was produced at a larger rate than benzo[c]carbazole, and the content gradually exceeded benzo[c]carbazole. Molecular mechanics data on these two benzocarbazoles indicate that benzo[a]carbazole is more unstable than benzo[c]carbazole, thus indicating that the prominence of benzo[a]carbazole is caused by specific precursors within the kerogen^16^. The content of benzo[a]carbazole, benzo[b]carbazole and benzo[c]carbazole reached their maximum at 1.48%*R*_*o*_, with maximum values of 158.62 μg/g, 75.37 μg/g and 121.29 μg/g, respectively. With the maturity continued to increase, the content of benzocarbazole isomers rapidly decreased. The content of all three isomers were below 3 μg/g at 2.26%*R*_*o*_. The relative abundance of benzo[a]carbazole and benzo[c]carbazole was greatly influenced by maturity, ranging from 30 to 50% and 31 to 54%, respectively. With the increasing maturity, the relative abundance of benzo[a]carbazole continued to increase (Fig. 11), while the relative abundance of benzo[c]carbazole continued to decrease (Table 3). The relative abundance of benzo[b]carbazole showed a trend of first increasing and then decreasing with the increasing maturity, ranging from 15 to 31%, with maximum at 1.11%*R*_*o*_.

### Changes in parameters of carbazole compounds with the increasing maturity

According to the migration and fractionation mechanism of carbazole compounds, the concentration changes of carbazole compounds have been widely applied in hydrocarbon migration. The above has proven that thermal maturity has a significant impact on the distribution of carbazole compounds in coal source rocks. Coincidentally, this study also found that thermal maturity also affects the ratio of carbazole compounds, but the degree of influence varies. When applying parameters of carbazole compounds with significant differences in maturity, it is necessary to treat them differently and fully consider the impact of thermal maturity.

#### Methylcarbazole isomers

**Figure 12:**
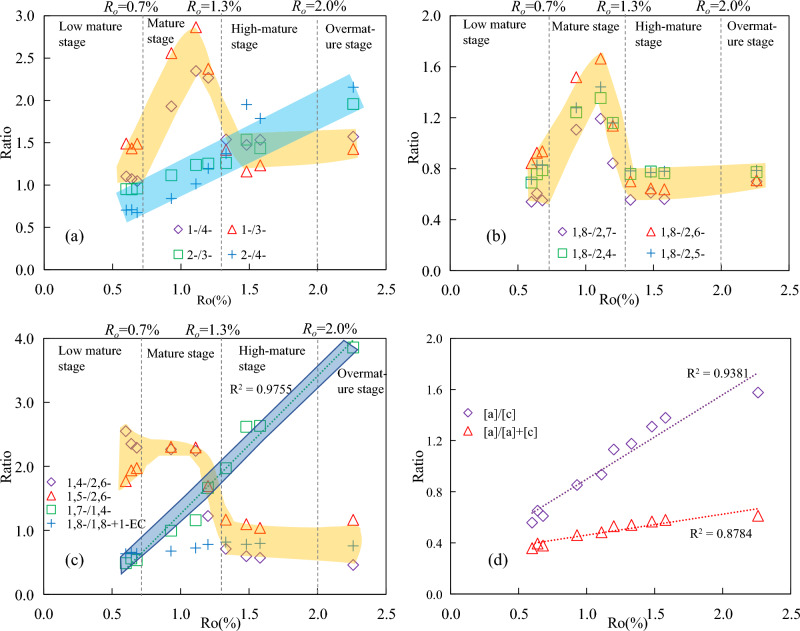
Changes in parameters of carbazole compounds with the increasing maturity. (**a**) methylcarbazole isomers. (**b**,**c**) dimethylcarbazole isomers. (**d**) benzocarbazole isomers. 1-/4-:1-methylcarbazole/4-methylcarbazole;1,8-/2,7-:1,8-dimethylcarbazole/2,7-dimethylcarbazole; [a]/[c]:benzo[a]carbazole/benzo[c]carbazole;[a]/[a] + [c]:benzo[a]carbazole/benzo[a]carbazole + benzo[c]carbazole.

In the methylcarbazole isomers, with the increasing maturity, the ratios between the isomers showed different changes (Fig. 12). In the mature stage, 1-/4-MCA and 1-/3-MCA undergo wide range of 1.4–3.0, with a pattern of first increasing and then decreasing. The maximum appeared around 1.11%*R*_*o*_, and the ratio remained basically unchanged after 1.5%*R*_*o*_. 2-/3-MCA and 2-/4-MCA linearly increased with the increasing maturity and showed a good correlation with maturity, which may be potential maturity indicators (Fig. 12a).

#### Dimethylcarbazole isomers

In the dimethylcarbazole isomers, with the increasing maturity, the ratios of the content of shielded isomers (1,8-DMCA) to the content of exposed isomers (2,7-DMCA, 2,6-DMCA, et al.) showed a similar variation pattern (Fig. 12b). In the mature stage, the ratios all showed a significant change of first increasing and then decreaing, ranging from 0.7 to 1.8 (maximum at 1.11%*R*_*o*_*)*. After *R*_*o*_ > 1.5%, the ratios remained almost unchanged. The ratio of the semi-shielded isomer to the exposed isomer is such as 1,4-/2,6-DMCA, and the ratio gradually decreased from 2.55 to 0.46 with the increasing maturity. 1,5-/2,6-DMCA also exhibits a significant increase and then decrease in the mature stage, while the ratios remained basically unchanged after *R*_*o*_ > 1.5%. The ratio between semi shielded isomers, such as 1,7-/1,4-DMCA linearly increased from 0.49 to 3.8 with the increasing maturity. 1, 8-/1,8-+1-EC increased with the increasing maturity, with limit range between 0.60 and 0.82(Fig. 12c). The dealkylation of 1-ethylcarbazole was suggested to have an equivalent mechanism to 4-ethyldibenzothiophene^43^, as the dealkylation of 1-ethylcarbazole leads to the formation of 1-methylcarbazole. If dealkylation occurs, an increase for this ratio can be reasonably expected.

#### Benzocarbazole isomers

In the benzocarbazole isomers, maturity had a great influence on the abundance of benzocarbazole in source rocks and isomers ratios, but relatively limited influence on [a]/[a] + [c]. [a]/[a] + [c] and [a]/[c] gradually increased with the increasing maturity, [a]/[c] showed a large range of changes from 0.56 to 1.58. Clegg et al.^13^ previously reported that the benzocarbazole a/c ratio was initially low and then rose rapidly in the classical Posidonia Shale maturation series from the Hils Syncline, Germany (0.48–1.45% Rr). Harrison et al.^16^ observed a similar feature for the Hekkingen Formation, Norway and Westphalian coals, Germany (0.4–1.0% Rr) and Li et al.^17^ documented a strong maturation influence for the Duvernay Formation, Canada. [a] /[a] + [c] only varies within a relatively small range of 0.36–0.61, showing limited maturity dependence (Fig. 12d), which is similar to the findings of some scholars who believe that maturity has a certain impact on the distribution of carbazole in source rocks, but has a very limited impact on [a]/[a] + [c]^13,15,17,18,20,21^. Studies have shown that [a]/[c] can be used as tracers for oil–gas migration, and their absolute concentrations in crude oil decrease with the increasing migration distance^45,46^. Therefore, the changes that occur with the increasing maturity [a]/[c] are opposite to the changes that occur with migration distance.

## Geological significances

During the hydrocarbon migration, nitrogen-containing compounds will undergo migration fractionation effect. Therefore, the concentration and ratios of carbazole compounds can be used as indicators of hydrocarbon migration. The experimental data in this article showed that thermal maturity controls the generation of a large amount of carbazole compounds in coal source rocks. Therefore, when using carbazole compounds to determine the oil and gas migration path in coal bearing strata, it is necessary to fully consider the impact of thermal maturity on carbazole concentration to avoid the migration fractionation effect masking the true maturity effect.

Yunlai Yang and Khaled Arouri^47^ reported a new method based on correlation analysis of geological tracer components: by analyzing the initial carbazole concentrations of source rocks in five oil fields in Saudi Arabia and the correlation coefficients of each concentration, the filling sequence and migration path of oil are revealed, and the migration path and filling relationship obtained by comparing the carbazole components and the basin simulation analysis show that the conclusions obtained by the two methods are basically the same. Sandu et al.^48^ used carbazole compounds as tracers and an internal tracer composition simulator to calculate the relationship between tracer composition and volume. Then, an envelope function was constructed to estimate the initial geological reserves of oil and gas. However, the initial carbazole concentration in the source rock is affected by the type and maturity of the source rock. The greater the influence of the type and maturity of the source rock, the greater the range of carbazole composition correlation coefficient in different regions, and the more decisive the migration path will be. In this paper, it has been found that carbazole in coal source rocks is significantly affected by maturity. When this method is used to judge the oil and gas migration path in areas with large differences in maturity, the difference in carbazole composition correlation coefficient will be more obvious. Therefore, the conclusion that thermal maturity controls the abundant generation of carbazole in coal source rocks provides a good theoretical basis for the subsequent use of this method for oil and gas migration path analysis and basin modeling.

## Conclusions

1. The thermal maturity has a significant impact on the content distribution of carbazole compounds, especially in the mature and high mature stages. Compared with the low mature stage, the content of carbazole compounds was about 10–100 times higher in the mature stage. At over mature stage with *R*_*o*_ higher than 2.5%, almost no carbazole compounds could be detected. The main formation stage of carbazoles is later than the stage of hydrocarbon generation, but its formation curve is close to that of hydrocarbon generation. It can be seen that carbazole compounds may not be completely inherited from organic sediments, and their content increases significantly with the appearance of the main hydrocarbon generation period. Therefore, thermal maturity significantly controls the large generation of carbazole in coal source rocks.
2. In coal source rocks, the relative abundance of carbazole isomers exhibits different variation laws. With the increasing maturity, the relative abundance of 2-MCA, 1,7-DMCA, B[a]CA gradually increased, while the relative abundance of 4-MCA, 1,4-DMCA, B[a]CA gradually decreased. The relative abundance of 1,8-DMCA and B[b]CA showed a relatively significant increasing and then decreasing during the mature stage, reached its maximum at 1.11%*R*_*o*_. The relative abundance of 2,7-DMCA, 2,6-DMCA, 2,5-DMCA, and 2,4-DMCA showed a relatively significant decreased and then increased in the mature stage, and also reached its maximum at 1.11%*R*_*o*_. The relative abundance of 2,5-DMCA remains basically unchanged throughout the maturity stage.
3. The ratio of carbazole compounds in coal source rocks is influenced by maturity differently. The ratio of [a]/[c] shows a good correlation with maturity as maturity increases, or can become a maturity indicator; while [a]/[a] + [c] only changes in the range of 0.36–0.61 in the entire maturity range, showing limited maturity dependence. The limited data presented here further confirm earlier conclusions of maturation effect on carbazole distributions in coal source rocks and extend the maturation range into high-maturity stage and over-maturity stage.
4. Of course, the experimental results of this article also have certain limitations, mainly including the following two aspects: (1) The results obtained from laboratory simulation experiments may differ from the actual geological conditions. When applying laboratory results to geological conditions, caution should be exercised and the influence of other factors should be comprehensively considered; (2) The experiment only studied the effect of thermal maturity on the distribution of carbazole compounds in coal. It is uncertain whether thermal maturity has the same effect on the distribution of carbazole compounds in other types of source rocks, and further experiments are needed to prove this. In future research, the author will conduct thermal simulation experiments on different types of source rocks to analyze the impact of thermal maturity on carbazole compounds in different types of source rocks. And in this experiment, it was found that the content and ratio of some compounds have a good correlation with *R*_*o*_. In the future, simulation experiments will be conducted for different types of source rocks, and effective maturity indicators may be found in non-hydrocarbon compounds.
